# Characterization of the complete mitochondrial genome of *Cephalopholis miniata* (Perciformes, Serranidae) and its phylogenetic analysis

**DOI:** 10.1080/23802359.2021.1937363

**Published:** 2021-06-14

**Authors:** Lei Meng, Ying Zhang, Li Gong, Yang Gao

**Affiliations:** a Fishery School of Zhejiang Ocean University, Laboratory of Aquatic Animal Genetics and Development, Zhoushan, China; b School of Marine Science and Technology of Zhejiang Ocean University, National and Provincial Joint Laboratory of Exploration and Utilization of Marine Aquatic Genetic Resources, Zhoushan, China

**Keywords:** Cephalopholis miniata, Epinephelinae, mitogenome, phylogenetic analysis

## Abstract

*Cephalopholis miniata* belongs to the family Serranidae of Perciformes, which is one of the most important species in coral-reef ecosystem. In this study, its complete mitochondrial genome is presented. The assembled mitogenome is 16,585 bp and includes 13 protein-coding genes (PCGs), 22 transfer RNA genes (tRNAs), two ribosomal RNA genes (12S and 16S), and an AT-rich region (also called control region, CR). The overall base composition is 29.2% A, 26.1% T, 16.2% G, and 28.5% C, with an A + T bias of 55.3%. Except *ND6* and eight tRNAs, all other mitochondrial genes are encoded on the heavy strand. Phylogenetic analysis shows that all species of the genus *Cephalopholis* cluster into a clade, including the newly sequenced mitogenome of *C. miniata*.

Groupers are bottom-associated fishes found in the tropical and subtropical waters of all oceans. The coral hind, *Cephalopholis miniata* (Forsskål 1775), is a kind of groupers belonging to the family Serranidae of Perciformes. It is widespread throughout the tropical waters of the Indo-West Pacific area, including Durban, South Africa, the Red Sea, and Line Islands (Rocha 2018). *C. miniata* is an important species in commercial fisheries at the local level. Meanwhile, it shows important ecological functions because it is one of the major predators feeding on a variety of fishes, crustaceans, and cephalopods in coral-reef ecosystem (Randall and Brock 1960; Heemstra and Randall 1993; Pinault et al. 2014). In this study, we described the complete mitochondrial genome (mitogenome) of *C. miniata* and explored the phylogenetic relationship within Serranidae. All the results presented in this study would provide insights into the evolution and conservation genetics of the genus *Cephalopholis*.

Sample of *C. miniata* was collected from Sansha, Hainan Province of China (112°22′40″E, 16°50′25″N) and stored in laboratory of Zhejiang Ocean University with accession number HNSS021. Total genomic DNA was extracted using a phenolch: loroform extraction protocol (Sambrook and Russell 2006). Based on the existing complete mitogenome of *Cephalopholis urodeta* (NC_030057), 12 pairs of primers were designed. The samples were amplified by PCR, and then sequenced using Sanger sequencing technology. Annotation of the complete mitogenome sequence was performed using Sequin (version 15.10, http://www.ncbi.nlm.nih.gov/Sequin/) and tRNAscan-SE 1.21 (Lowe and Chan 2016). The complete mitogenome sequence of *C. miniata* was deposited into GenBank database with the accession number MW423580.

The complete mitogenome of *C. miniata* was a closed-circular molecule of 16,585 bp in length. It contained 13 protein-coding genes (PCGs), two ribosomal RNA genes (12S and 16S), 22 transfer RNA genes (tRNA), and a putative control region (CR), which was the same as other Perciformes species (Ceruso et al. 2018; Mascolo et al. 2018; Ceruso et al. 2020). The overall base composition was A (29.2%), T (26.1%), G (16.2%), C (28.5%); respectively, with a slight AT bias (55.3%). Except one PCGs (*ND6*) and eight tRNAs (*tRNA-Gln*, *Ala*, *Asn*, *Cys*, *Tyr*, *Ser*^AGT^, *Glu*, and *Pro*), which were distributed on the light (L-) strand, the rest of mitochondrial genes were distributed on the heavy (H-) strand. The total length of 13 PCGs was 11,406 bp. All of the 13 PCGs were initiated by the start codon ATN (ATA and ATG), with an exception (GTG) in *COI*. The majority of the 13 PCGs terminated with TAA or TAG, while three other PCGs (*COII*, *ND4*, and *Cyt b*) used a single T as a stop codon. The mitogenome of *C. miniata* contained 22 tRNA genes scattered throughout the genome. Most tRNAs displayed a typical cloverleaf secondary structure except for *tRNA-Ser* (GCT) that lacked the dihydrouridine (DHU) arm. The *12S* and *16S rRNA* genes were located between the *tRNA-Phe* and *tRNA-Leu* (TAA) genes, and were separated by the *tRNA-Val* gene. The CR of *C. miniata* was located between *tRNA-Phe* and *tRNA-Pro*, with a total length of 878 bp.

To ascertain the phylogenetic position of *C. miniata*, we downloaded 17 mitogenomes of Serranidae species and two outgroup species (*Mastacembelus favus* and *Monopterus albus*) from GenBank to conduct phylogenetic analysis (Figure 1). The Bayesian Inference (BI) tree was constructed using the software MrBayes 3.2.6 (Ronquist et al. 2012) based on the nucleotide sequences of 12 PCGs (except *ND6*). The GTR + F + I + G4 model selected by ModelFinder (Kalyaanamoorthy et al. 2017) was applied in the BI analysis. The results showed that all species of the genus *Cephalopholis* clustered into a clade, including the newly sequenced mitogenome of *C. miniata*, indicating the monophyly of this genus. We expect the present results will provide an important dataset for phylogenetic and taxonomic analyses of genus *Cephalopholis* species.

**Figure 1.**
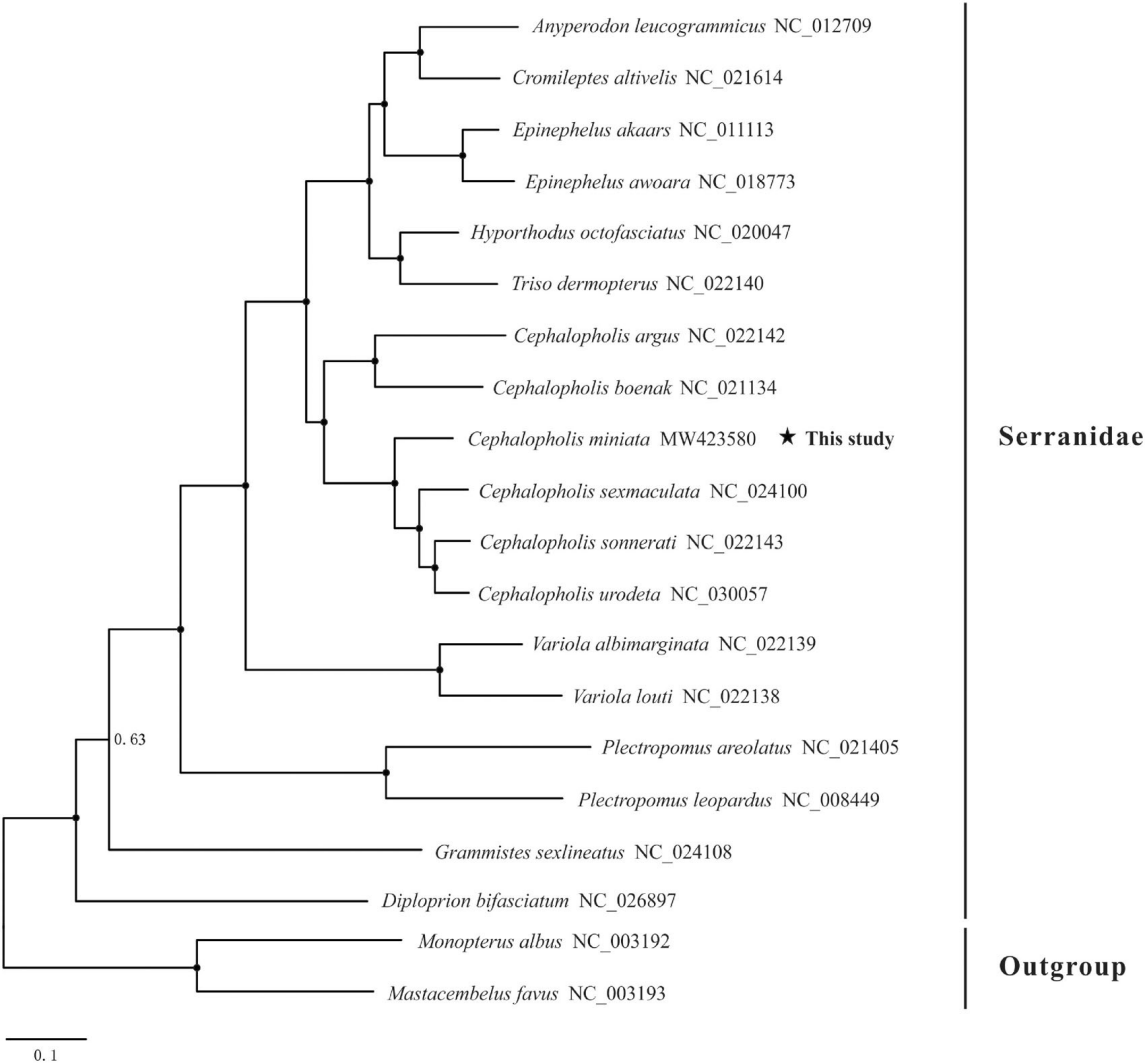
Phylogenetic tree of Serranidae species inferred from the 12 PCGs based on Bayesian inference (BI) analysis. Values at the branches represent Bayesian posterior probabilities (BPP). Node marked with solid circle indicates 100% supporting value. The number after the species name is the GenBank accession number.

## Data Availability

Mitochondrial genome sequence can be accessed via accession number MW423580 (https://www.ncbi.nlm.nih.gov/nuccore/MW423580) in the NCBI GenBank.
